# Supplementary material for: Chiral Ag23 nanocluster with open shell electronic structure and helical face-centered cubic framework
Source: Nat Commun. 2018 Feb 21;9:744. doi: 10.1038/s41467-018-03136-9 (PMC5821857; doi:10.1038/s41467-018-03136-9)
Supplement: Supplementary file 1 — Supplementary Information [file 41467_2018_3136_MOESM1_ESM.pdf]

Supplementary Information

Chiral Ag_{23} Nanocluster with Open Shell Electronic Structure and Helical Face-Centered Cubic Framework

Chao Liu,[1] Tao Li,[2] Hadi Abroshan,[3] Zhimin Li,[1] Chen Zhang,[4] Hyung J. Kim,[3,5] Gao Li[1] & Rongchao Jin[3]

[1] State Key Laboratory of Catalysis, Dalian Institute of Chemical Physics, Chinese Academy of Sciences, Dalian 116023, P. R. China

[2] School of Physical Science and Technology, ShanghaiTech University, Shanghai 201210, P. R. China

[3] Department of Chemistry, Carnegie Mellon University, Pittsburgh, Pennsylvania 15213, United States

[4] Department of Chemistry, University of Missouri-Columbia, Columbia, Missouri 65211, United States

[5] School of Computational Sciences, Korea Institute for Advanced Study, Seoul 02455, Korea

*Correspondence and requests for materials should be addressed to:

gaoli@dicp.ac.cn (G.L.); rongchao@andrew.cmu.edu (R.J.)

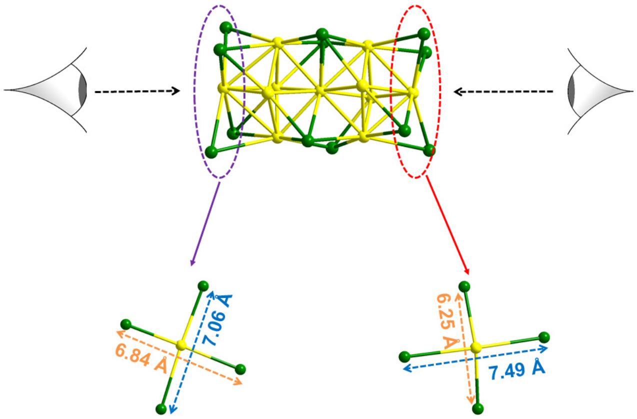

Supplementary Figure 1. Deformation of the skeleton' ends from a square shape to rhombus like arrangements due to twist of cells relative to each other by ~ 27°about the longitudinal axis of the supercell.

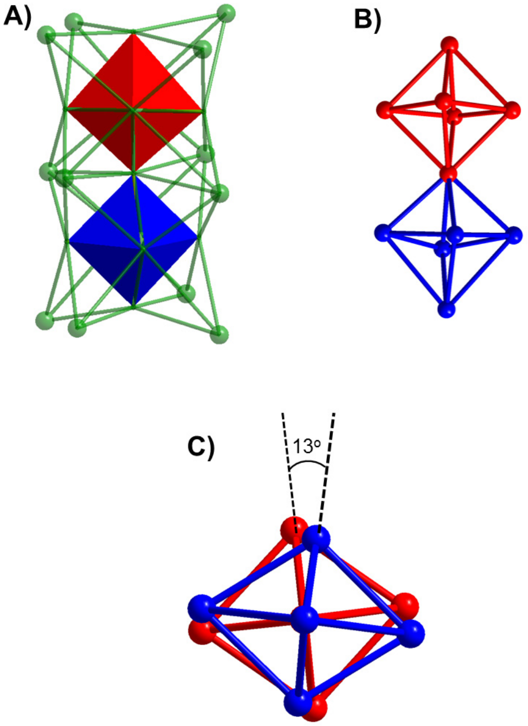

Supplementary Fig. 2 (A) Two rhombic bipyramids composed of 11 silver atoms located at the face centers are highlighted by blue and red facets. (B) The silver atoms building the bipyramids are shown, while other atoms are omitted for clarity. (C) Two bipyramids are found to be twisted relative to each other by ~ 13°.

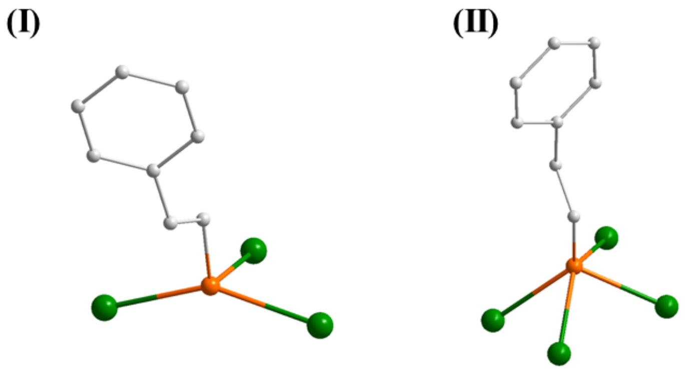

Supplementary Fig. 3 Two main categories of thiolate ligands of $Ag_{23}(PPh_3)_8(SC_2H_4Ph)_{18}$ cluster: (I) tri- and (II) tetra-podal fashions of binding pattern with the surface Ag atoms. Color labels: Ag, green; S, orange; C, grey. All hydrogen atoms are omitted for clarity.

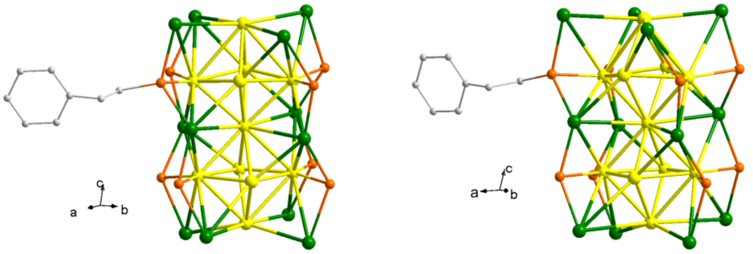

Supplementary Fig. 4 Top and side views of binding of thiolate ligands (subcategories IA with tri-podal type) with the surface Ag atoms of the nanocluster. Of note, only –C_2H_4Ph tail of one of the thiolate ligands is shown for clarity.

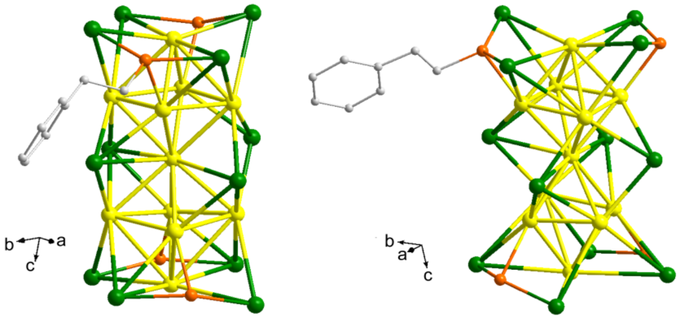

Supplementary Fig. 5 Top and side views of binding of thiolate ligands (subcategories IB with tri-podal type) with the surface Ag atoms of the nanocluster. Of note, only –C_2H_4Ph tail of one of the thiolate ligands is shown for clarity.

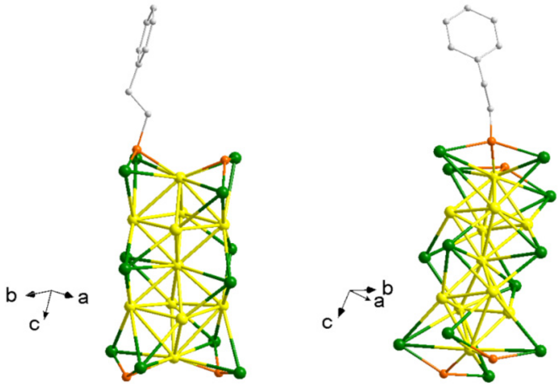

Supplementary Fig. 6 Top and side views of binding of thiolate ligands (subcategories IC with tri-podal type) with the surface Ag atoms of the nanocluster. Of note, only –C_2H_4Ph tail of one of the thiolate ligands is shown for clarity.

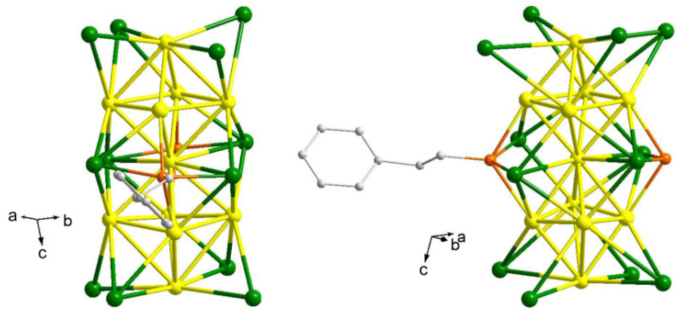

Supplementary Fig. 7 Top and side views of binding of thiolate ligands (categories II with tetra-podal type) with the surface Ag atoms of the nanocluster. Of note, only –C_2H_4Ph tail of one of the thiolate ligands is shown for clarity.

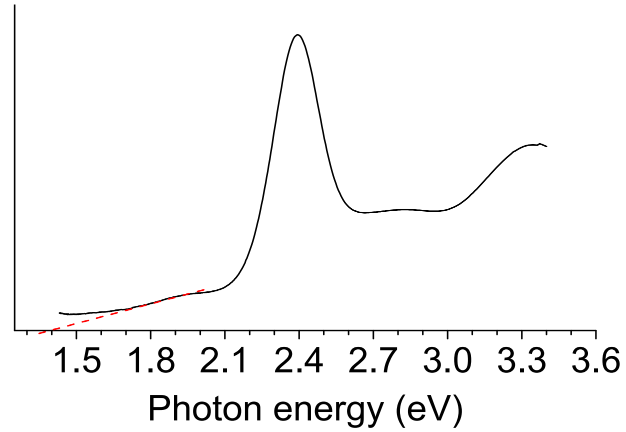

Supplementary Fig. 8 Spectrum on the energy scale (eV), the HOMO–LUMO gap is ca. 1.4 eV.

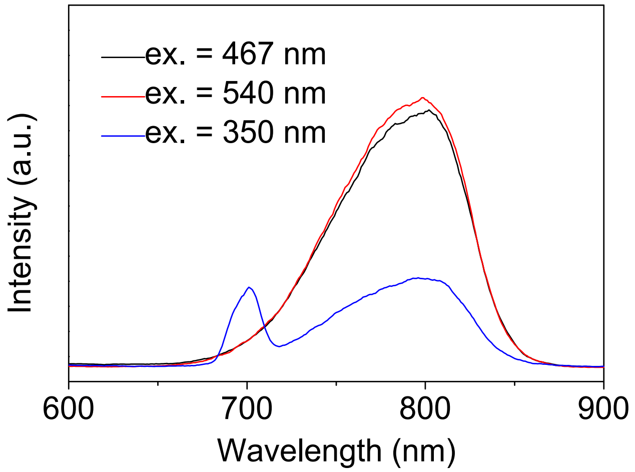

Supplementary Fig. 9 Photoluminescence spectra of $Ag_{23}(PPh_3)_8(SC_2H_4Ph)_{18}$ nanoclusters using different excitation wavelengths (350, 467, and 540 nm).

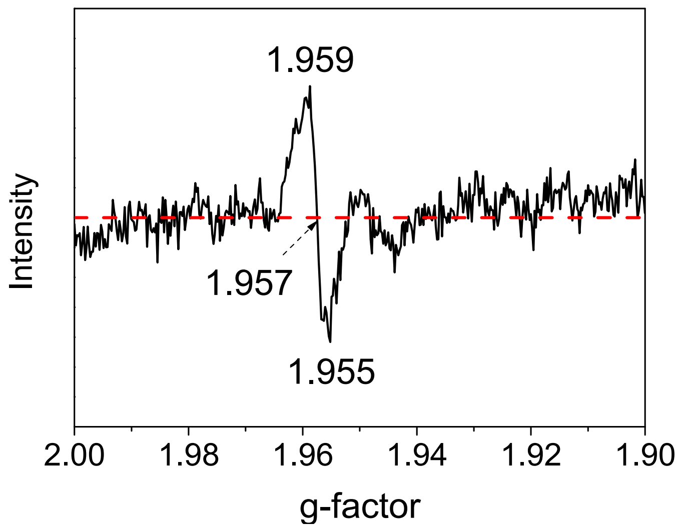

Supplementary Fig. 10 EPR spectrum of the $Ag_{23}(PPh_3)_8(SC_2H_4Ph)_{18}$ nanoclusters.

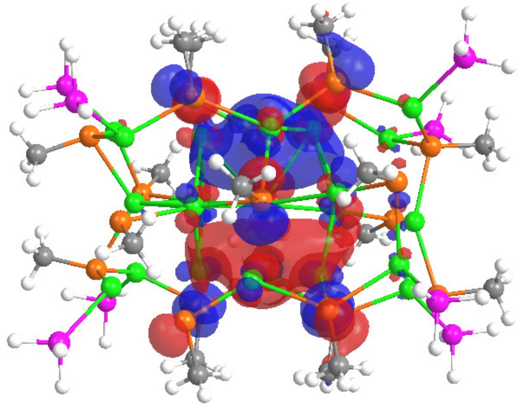

Supplementary Fig. 11 Schematic diagrams of α-HOMO of $Ag_{23}(PH_3)_8(SCH_3)_{18}$. Color labels: Ag, green; S, orange; P, purple; C, grey; H, white.

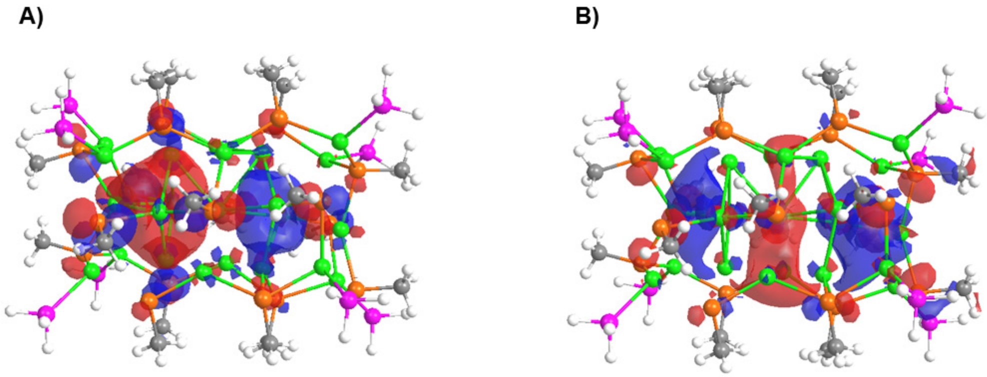

Supplementary Fig. 12 Schematic diagrams of (A) α-HOMO-1 and (B) α-LUMO of $Ag_{23}(PH_3)_8(SCH_3)_{18}$. Color labels: Ag, green; S, orange; P, purple; C, grey; H, white.

Supplementary Table 1 Comparison of the Ag-Ag distances and the twist angles of the Ag_{23} cluster in experiment and theory.

distances and angles	experiment	theory
Ag-Ag in bipyramid	2.894 Å (2.754-3.295 Å)	2.933 Å (2.863-3.071 Å)
Ag-S (tetra-podal type)	2.635 Å	2.763Å
	(Ag-SC_2H_4Ph: 2.589-2.718 Å)	(Ag-SCH_3: 2.636-3.127Å)
Ag-S (tri-podal type)	2.551 Å	2.641 Å
	(Ag-SC_2H_4Ph: 2.394-2.711 Å)	(Ag-SCH_3: 2.526-2.783 Å)
Ag-P	2.462 Å	2.620 Å
	(Ag-PPh_3: 2.412-2.506 Å)	(Ag-PH: 2.540-2.689 Å)
twist of cell (Ag_{14} unit)	27°	32°
twist of bipyramid	13°	17°

Supplementary Note 1

Experimental for X-ray crystallographic determination

Single X-ray diffraction data of Ag_{23} were collected on a Bruker X8 Prospector Ultra equipped with an Apex II CCD detector and an IμS micro-focus CuK\α X-ray source (λ = 1.54178 nm). A piece of brown square plate crystal with dimensions 0.2 × 0.2 × 0.05 mm was mounted onto a MiTeGen micromount with fluorolube under a constant flow of cold nitrogen gas. The data were collected at 100 K. A monoclinic unit cell with dimensions a = 38.6694(11), b = 38.7589(13), c = 45.4061(13), α = 90 °, β = 108.033(2) °, and γ = 90 ° was derived from the least-square refinement of 9967 reflections in the range of 2.326 < θ < 47.151. Non-centrosymmetric space group *Cc* was determined based on systematic absences.

The data were only collected to 0.9 Å with four fold redundancy and a completeness of 98.5%. After integration of the data by Bruker program SAINT, empirical absorption correction was applied using program SADABS.[1]

The structure was successfully solved by direct method using Bruker program SHELXTL.[2] All 23 Ag atoms, 18 S atoms, 8 P atoms, and some of the C atoms were located easily. Although most of the C atoms were generated via subsequent difference Fourier syntheses, some of the carbon atoms still could not be found. A total of 519 restraints were applied to optimize atomic displacement parameters of the carbon atoms. The highest residual Q peak has a peak height of 2.93 and other 61 Q peaks fall between 1 and 2. Some of them may be attributed to disordered solvent molecules. All the Ag, S, and P atoms are refined anisotropically. However, anisotropic refinement of C atoms leads to inability of convergence. Therefore, all carbon atoms were isotropically refined. Due to the same reason, idealized H atom positions were not assigned.

CCDC-1811890 contain the supplementary crystallographic data for $Ag_{23}(SR)_{18}(PPh_3)_8$ in this paper. These data can be obtained free of charge from Cambridge Crystallographic Data Centre via www.ccdc.cam.ac.uk/data_request/cif.

Supplementary Table 2 Crystal data and structure refinement for $Ag_{23}(SR)_{18}(PPh_3)_8$.

Identification code	$Ag_{23}(SR)_{18}(PPh_3)_8$	
Empirical formula	C576 H564 Ag46 P16 S36	
Formula weight	14097.94	
Temperature	100(2) K	
Wavelength	1.54178 Å	
Crystal system	Monoclinic	
Space group	Cc	
Unit cell dimensions	a = 38.6694(11) Å	α= 90°.
	b = 38.7589(13) Å	β= 108.033(2)°.
	c = 45.4061(13) Å	γ = 90°.
Volume	64711(3) $Å^3$	
Z	4	
Density (calculated)	1.447 Mg/m^3	
Absorption coefficient	12.674 mm^{-1}	
F(000)	27992	
Crystal size	0.200 x 0.200 x 0.050 mm^3	
Theta range for data collection	2.046 to 59.176°.	
Index ranges	-42<=h<=42, -41<=k<=42, -50<=l<=50	
Reflections collected	181820	
Independent reflections	83303 [R(int) = 0.1034]	
Completeness to theta = 59.176°	98.5 %	
Absorption correction	Semi-empirical from equivalents	
Max. and min. transmission	1 and 0.603	
Refinement method	Full-matrix least-squares on F^2	
Data / restraints / parameters	83303 / 519 / 2079	
Goodness-of-fit on F^2	1.011	
Final R indices [I>2sigma(I)]	R1 = 0.1172, wR2 = 0.3003	
R indices (all data)	R1 = 0.1803, wR2 = 0.3479	
Absolute structure parameter	0.911(7)	
Extinction coefficient	n/a	
Largest diff. peak and hole	2.933 and -1.720 e.$Å^{-3}$	

Supplementary Table 3 Bond lengths [Å] and angles [°] for $Ag_{23}(SR)_{18}(PPh_3)_8$.

P(1)-Ag(23)	2.451(15)	P(2)-Ag(13)	2.471(16)
P(3)-Ag(14)	2.468(11)	P(4)-Ag(21)	2.432(14)
P(5)-Ag(22)	2.482(13)	P(6)-Ag(16)	2.433(13)
P(7)-Ag(44)	2.488(12)	P(8)-Ag(45)	2.415(15)
P(9)-Ag(40)	2.506(14)	P(10)-Ag(29)	2.421(14)
P(11)-Ag(36)	2.463(12)	P(12)-Ag(35)	2.455(15)
P(13)-Ag(37)	2.488(12)	P(14)-Ag(12)	2.467(14)
P(15)-Ag(20)	2.496(16)	P(16)-Ag(46)	2.474(15)
S(1)-Ag(32)	2.500(15)	S(1)-Ag(45)	2.592(13)
S(1)-Ag(40)	2.620(14)	S(2)-Ag(7)	2.525(13)
S(2)-Ag(13)	2.572(15)	S(2)-Ag(23)	2.611(15)
S(3)-Ag(5)	2.426(14)	S(3)-Ag(8)	2.521(13)
S(3)-Ag(23)	2.651(14)	S(7)-Ag(36)	2.615(13)
S(4)-Ag(41)	2.479(13)	S(8)-Ag(4)	2.474(13)
S(4)-Ag(39)	2.528(14)	S(8)-Ag(16)	2.567(13)
S(4)-Ag(35)	2.616(14)	S(8)-Ag(22)	2.659(13)
S(5)-Ag(18)	2.471(16)	S(9)-Ag(38)	2.538(12)
S(5)-Ag(13)	2.595(12)	S(9)-Ag(37)	2.612(12)
S(5)-Ag(14)	2.635(12)	S(9)-Ag(35)	2.637(14)
S(6)-Ag(42)	2.480(16)	S(10)-Ag(17)	2.406(12)
S(6)-Ag(36)	2.590(13)	S(10)-Ag(10)	2.530(12)
S(6)-Ag(37)	2.629(13)	S(10)-Ag(20)	2.751(12)
S(7)-Ag(31)	2.463(12)	S(11)-Ag(28)	2.497(11)
S(7)-Ag(30)	2.533(11)	S(11)-Ag(40)	2.575(14)
S(11)-Ag(29)	2.610(14)	Ag(1)-Ag(5)	3.105(5)
S(12)-Ag(44)	2.548(15)	Ag(1)-Ag(19)	3.119(5)
S(12)-Ag(26)	2.555(13)	Ag(2)-Ag(7)	2.818(4)
S(12)-Ag(45)	2.667(14)	Ag(2)-Ag(9)	2.831(4)
S(13)-Ag(31)	2.422(12)	Ag(2)-Ag(6)	2.838(4)
S(13)-Ag(25)	2.523(12)	Ag(2)-Ag(3)	2.848(4)
S(13)-Ag(44)	2.649(13)	Ag(2)-Ag(11)	2.952(4)
S(14)-Ag(3)	2.503(12)	Ag(2)-Ag(8)	2.967(4)
S(14)-Ag(20)	2.568(14)	Ag(2)-Ag(10)	3.001(4)
S(14)-Ag(16)	2.607(15)	Ag(2)-Ag(15)	3.225(4)
S(15)-Ag(33)	2.425(11)	Ag(2)-Ag(5)	3.312(4)
S(15)-Ag(30)	2.518(13)	Ag(2)-Ag(17)	3.376(5)
S(15)-Ag(37)	2.615(12)	Ag(3)-Ag(10)	2.813(4)
S(16)-Ag(15)	2.436(11)	Ag(3)-Ag(4)	2.813(5)
S(16)-Ag(11)	2.522(11)	Ag(3)-Ag(17)	3.024(5)
S(16)-Ag(14)	2.607(12)	Ag(3)-Ag(16)	3.303(5)
S(17)-Ag(9)	2.500(12)	Ag(4)-Ag(9)	2.800(4)
S(17)-Ag(22)	2.603(12)	Ag(4)-Ag(10)	3.020(5)
S(17)-Ag(21)	2.604(12)	Ag(4)-Ag(22)	3.164(5)

S(18)-Ag(17)	2.454(11)	Ag(4)-Ag(20)	3.220(6)
S(18)-Ag(11)	2.530(11)	Ag(5)-Ag(7)	3.014(4)
S(18)-Ag(13)	2.635(13)	Ag(5)-Ag(8)	3.074(5)
S(19)-Ag(26)	2.578(11)	Ag(6)-Ag(8)	2.744(5)
S(19)-Ag(43)	2.608(12)	Ag(6)-Ag(11)	2.789(4)
S(19)-Ag(27)	2.641(12)	Ag(6)-Ag(18)	2.831(5)
S(19)-Ag(31)	2.669(13)	Ag(6)-Ag(15)	3.051(4)
S(20)-Ag(19)	2.464(12)	Ag(6)-Ag(12)	3.259(5)
S(20)-Ag(8)	2.507(14)	Ag(7)-Ag(11)	2.776(4)
S(20)-Ag(12)	2.637(11)	Ag(7)-Ag(8)	2.787(5)
S(21)-Ag(41)	2.419(13)	Ag(7)-Ag(18)	2.815(5)
S(21)-Ag(34)	2.487(12)	Ag(7)-Ag(13)	3.161(5)
S(21)-Ag(40)	2.713(13)	Ag(8)-Ag(19)	3.062(5)
S(22)-Ag(6)	2.518(10)	Ag(8)-Ag(18)	3.365(6)
S(22)-Ag(12)	2.607(11)	Ag(9)-Ag(10)	2.793(5)
S(22)-Ag(14)	2.642(11)	Ag(9)-Ag(19)	3.030(5)
S(23)-Ag(3)	2.580(11)	Ag(10)-Ag(15)	3.072(4)
S(23)-Ag(5)	2.635(12)	Ag(10)-Ag(17)	3.099(5)
S(23)-Ag(7)	2.661(10)	Ag(11)-Ag(17)	3.057(4)
S(23)-Ag(17)	2.669(12)	Ag(11)-Ag(15)	3.073(4)
S(24)-Ag(19)	2.435(11)	Ag(11)-Ag(18)	3.272(6)
S(24)-Ag(1)	2.523(13)	Ag(14)-Ag(18)	3.360(5)
S(24)-Ag(22)	2.670(13)	Ag(24)-Ag(26)	2.842(5)
S(25)-Ag(33)	2.589(12)	Ag(24)-Ag(38)	2.842(5)
S(25)-Ag(28)	2.591(12)	Ag(24)-Ag(28)	2.847(4)
S(25)-Ag(38)	2.684(11)	Ag(24)-Ag(27)	2.855(4)
S(25)-Ag(41)	2.717(13)	Ag(24)-Ag(30)	2.951(4)
S(26)-Ag(9)	2.593(10)	Ag(24)-Ag(39)	2.956(4)
S(26)-Ag(15)	2.599(11)	Ag(24)-Ag(25)	2.980(4)
S(26)-Ag(19)	2.677(11)	Ag(24)-Ag(34)	3.003(4)
S(26)-Ag(6)	2.692(11)	Ag(24)-Ag(33)	3.274(5)
S(27)-Ag(5)	2.446(11)	Ag(24)-Ag(43)	3.276(5)
S(27)-Ag(1)	2.551(12)	Ag(25)-Ag(28)	2.769(5)
S(27)-Ag(16)	2.601(12)	Ag(25)-Ag(26)	2.818(5)
S(28)-Ag(33)	2.471(11)	Ag(25)-Ag(32)	3.033(5)
S(28)-Ag(25)	2.526(13)	Ag(25)-Ag(33)	3.077(5)
S(28)-Ag(29)	2.586(12)	Ag(25)-Ag(31)	3.135(5)
S(29)-Ag(15)	2.467(11)	Ag(26)-Ag(34)	2.802(5)
S(29)-Ag(10)	2.536(11)	Ag(26)-Ag(32)	2.805(5)
S(29)-Ag(21)	2.623(12)	Ag(26)-Ag(31)	3.031(5)
S(30)-Ag(43)	2.468(12)	Ag(26)-Ag(45)	3.368(5)
S(30)-Ag(34)	2.530(13)	Ag(27)-Ag(30)	2.753(5)
S(30)-Ag(45)	2.623(13)	Ag(27)-Ag(39)	2.797(5)
S(31)-Ag(4)	2.500(14)	Ag(27)-Ag(42)	2.806(6)
S(31)-Ag(21)	2.594(13)	Ag(27)-Ag(43)	3.040(5)

S(31)-Ag(20)	2.611(14)	Ag(27)-Ag(36)	3.223(4)
S(32)-Ag(18)	2.405(19)	Ag(28)-Ag(32)	2.811(5)
S(32)-Ag(23)	2.566(13)	Ag(28)-Ag(34)	2.817(5)
S(32)-Ag(12)	2.580(14)	Ag(28)-Ag(41)	3.022(5)
S(33)-Ag(32)	2.471(15)	Ag(28)-Ag(29)	3.352(5)
S(33)-Ag(29)	2.578(14)	Ag(30)-Ag(38)	2.800(5)
S(33)-Ag(44)	2.627(12)	Ag(30)-Ag(31)	3.037(5)
S(34)-Ag(43)	2.449(13)	Ag(30)-Ag(33)	3.080(4)
S(34)-Ag(39)	2.508(13)	Ag(30)-Ag(42)	3.241(7)
S(34)-Ag(46)	2.650(15)	Ag(32)-Ag(34)	3.036(5)
S(35)-C(515)	1.98(10)	Ag(32)-Ag(44)	3.202(5)
S(35)-Ag(42)	2.398(19)	Ag(32)-Ag(40)	3.210(5)
S(35)-Ag(46)	2.584(17)	Ag(33)-Ag(38)	3.019(5)
S(35)-Ag(35)	2.595(15)	Ag(34)-Ag(41)	3.089(5)
S(36)-Ag(27)	2.530(12)	Ag(34)-Ag(43)	3.112(5)
S(36)-Ag(36)	2.571(14)	Ag(35)-Ag(38)	3.217(5)
S(36)-Ag(46)	2.619(14)	Ag(38)-Ag(39)	2.762(5)
Ag(1)-Ag(3)	2.774(5)	Ag(38)-Ag(42)	2.838(6)
Ag(1)-Ag(9)	2.819(5)	Ag(39)-Ag(43)	3.072(5)
Ag(1)-Ag(2)	2.965(4)	Ag(39)-Ag(41)	3.129(5)
Ag(1)-Ag(4)	3.021(5)	Ag(39)-Ag(42)	3.294(6)
S(24)-Ag(1)-S(27)	120.4(4)	S(24)-Ag(22)-Ag(4)	97.1(3)
S(24)-Ag(1)-Ag(3)	160.1(3)	P(1)-Ag(23)-S(32)	111.7(5)
S(27)-Ag(1)-Ag(3)	76.9(3)	P(1)-Ag(23)-S(2)	124.7(6)
S(24)-Ag(1)-Ag(9)	79.8(3)	S(32)-Ag(23)-S(2)	104.5(5)
S(27)-Ag(1)-Ag(9)	159.4(3)	P(1)-Ag(23)-S(3)	116.4(5)
Ag(3)-Ag(1)-Ag(9)	82.47(13)	S(32)-Ag(23)-S(3)	107.1(4)
S(24)-Ag(1)-Ag(2)	117.0(3)	S(2)-Ag(23)-S(3)	89.6(4)
S(27)-Ag(1)-Ag(2)	109.5(3)	Ag(26)-Ag(24)-Ag(38)	171.35(15)
Ag(3)-Ag(1)-Ag(2)	59.39(11)	Ag(26)-Ag(24)-Ag(28)	80.50(12)
Ag(9)-Ag(1)-Ag(2)	58.54(10)	Ag(38)-Ag(24)-Ag(28)	103.00(13)
S(24)-Ag(1)-Ag(4)	104.2(3)	Ag(26)-Ag(24)-Ag(27)	102.27(14)
S(27)-Ag(1)-Ag(4)	110.0(3)	Ag(38)-Ag(24)-Ag(27)	75.24(13)
Ag(3)-Ag(1)-Ag(4)	57.89(11)	Ag(28)-Ag(24)-Ag(27)	172.66(15)
Ag(9)-Ag(1)-Ag(4)	57.18(11)	Ag(26)-Ag(24)-Ag(30)	113.83(13)
Ag(2)-Ag(1)-Ag(4)	91.17(13)	Ag(38)-Ag(24)-Ag(30)	57.78(11)
S(24)-Ag(1)-Ag(5)	122.9(3)	Ag(28)-Ag(24)-Ag(30)	128.71(14)
S(27)-Ag(1)-Ag(5)	50.1(3)	Ag(27)-Ag(24)-Ag(30)	56.59(11)
Ag(3)-Ag(1)-Ag(5)	75.06(13)	Ag(26)-Ag(24)-Ag(39)	129.01(14)
Ag(9)-Ag(1)-Ag(5)	124.23(14)	Ag(38)-Ag(24)-Ag(39)	56.85(12)
Ag(2)-Ag(1)-Ag(5)	66.08(11)	Ag(28)-Ag(24)-Ag(39)	115.46(14)
Ag(4)-Ag(1)-Ag(5)	132.77(16)	Ag(27)-Ag(24)-Ag(39)	57.50(12)
S(24)-Ag(1)-Ag(19)	49.8(3)	Ag(30)-Ag(24)-Ag(39)	93.64(13)
S(27)-Ag(1)-Ag(19)	133.6(3)	Ag(26)-Ag(24)-Ag(25)	57.84(12)

Ag(3)-Ag(1)-Ag(19)	126.84(14)	Ag(38)-Ag(24)-Ag(25)	117.33(14)
Ag(9)-Ag(1)-Ag(19)	61.12(11)	Ag(28)-Ag(24)-Ag(25)	56.69(10)
Ag(2)-Ag(1)-Ag(19)	68.50(11)	Ag(27)-Ag(24)-Ag(25)	130.54(14)
Ag(4)-Ag(1)-Ag(19)	116.41(14)	Ag(30)-Ag(24)-Ag(25)	88.46(12)
Ag(5)-Ag(1)-Ag(19)	93.89(12)	Ag(39)-Ag(24)-Ag(25)	170.36(15)
Ag(7)-Ag(2)-Ag(9)	171.00(16)	Ag(26)-Ag(24)-Ag(34)	57.22(11)
Ag(7)-Ag(2)-Ag(6)	73.98(12)	Ag(38)-Ag(24)-Ag(34)	131.33(14)
Ag(9)-Ag(2)-Ag(6)	103.00(13)	Ag(28)-Ag(24)-Ag(34)	57.50(11)
Ag(7)-Ag(2)-Ag(3)	103.10(13)	Ag(27)-Ag(24)-Ag(34)	118.18(13)
Ag(9)-Ag(2)-Ag(3)	80.96(12)	Ag(30)-Ag(24)-Ag(34)	169.81(15)
Ag(6)-Ag(2)-Ag(3)	172.16(15)	Ag(39)-Ag(24)-Ag(34)	89.62(13)
Ag(7)-Ag(2)-Ag(11)	57.46(10)	Ag(25)-Ag(24)-Ag(34)	89.93(12)
Ag(9)-Ag(2)-Ag(11)	128.40(13)	Ag(26)-Ag(24)-Ag(33)	116.27(13)
Ag(6)-Ag(2)-Ag(11)	57.55(10)	Ag(38)-Ag(24)-Ag(33)	58.65(11)
Ag(3)-Ag(2)-Ag(11)	114.68(13)	Ag(28)-Ag(24)-Ag(33)	70.35(11)
Ag(7)-Ag(2)-Ag(1)	117.18(13)	Ag(27)-Ag(24)-Ag(33)	113.72(13)
Ag(9)-Ag(2)-Ag(1)	58.15(11)	Ag(30)-Ag(24)-Ag(33)	59.03(10)
Ag(6)-Ag(2)-Ag(1)	130.87(14)	Ag(39)-Ag(24)-Ag(33)	114.68(13)
Ag(3)-Ag(2)-Ag(1)	56.96(11)	Ag(25)-Ag(24)-Ag(33)	58.70(11)
Ag(11)-Ag(2)-Ag(1)	170.14(14)	Ag(34)-Ag(24)-Ag(33)	127.84(13)
Ag(7)-Ag(2)-Ag(8)	57.54(12)	Ag(26)-Ag(24)-Ag(43)	70.62(11)
Ag(9)-Ag(2)-Ag(8)	113.63(14)	Ag(38)-Ag(24)-Ag(43)	113.89(13)
Ag(6)-Ag(2)-Ag(8)	56.36(11)	Ag(28)-Ag(24)-Ag(43)	116.48(13)
Ag(3)-Ag(2)-Ag(8)	128.49(14)	Ag(27)-Ag(24)-Ag(43)	58.96(11)
Ag(11)-Ag(2)-Ag(8)	94.67(13)	Ag(30)-Ag(24)-Ag(43)	114.68(12)
Ag(1)-Ag(2)-Ag(8)	88.07(12)	Ag(39)-Ag(24)-Ag(43)	58.80(11)
Ag(7)-Ag(2)-Ag(10)	131.81(14)	Ag(25)-Ag(24)-Ag(43)	128.45(14)
Ag(9)-Ag(2)-Ag(10)	57.14(10)	Ag(34)-Ag(24)-Ag(43)	59.23(10)
Ag(6)-Ag(2)-Ag(10)	118.94(13)	Ag(33)-Ag(24)-Ag(43)	171.72(13)
Ag(3)-Ag(2)-Ag(10)	57.41(10)	S(13)-Ag(25)-S(28)	120.6(4)
Ag(11)-Ag(2)-Ag(10)	89.06(12)	S(13)-Ag(25)-Ag(28)	160.6(3)
Ag(1)-Ag(2)-Ag(10)	89.85(11)	S(28)-Ag(25)-Ag(28)	77.0(3)
Ag(8)-Ag(2)-Ag(10)	169.76(14)	S(13)-Ag(25)-Ag(26)	79.8(3)
Ag(7)-Ag(2)-Ag(15)	114.85(12)	S(28)-Ag(25)-Ag(26)	159.2(3)
Ag(9)-Ag(2)-Ag(15)	69.43(10)	Ag(28)-Ag(25)-Ag(26)	82.26(13)
Ag(6)-Ag(2)-Ag(15)	60.01(10)	S(13)-Ag(25)-Ag(24)	115.8(3)
Ag(3)-Ag(2)-Ag(15)	116.21(13)	S(28)-Ag(25)-Ag(24)	110.1(3)
Ag(11)-Ag(2)-Ag(15)	59.46(10)	Ag(28)-Ag(25)-Ag(24)	59.22(11)
Ag(1)-Ag(2)-Ag(15)	127.57(13)	Ag(26)-Ag(25)-Ag(24)	58.61(11)
Ag(8)-Ag(2)-Ag(15)	115.12(12)	S(13)-Ag(25)-Ag(32)	105.7(3)
Ag(10)-Ag(2)-Ag(15)	58.99(9)	S(28)-Ag(25)-Ag(32)	109.1(3)
Ag(7)-Ag(2)-Ag(5)	58.23(10)	Ag(28)-Ag(25)-Ag(32)	57.73(11)
Ag(9)-Ag(2)-Ag(5)	116.80(12)	Ag(26)-Ag(25)-Ag(32)	57.16(11)
Ag(6)-Ag(2)-Ag(5)	112.47(13)	Ag(24)-Ag(25)-Ag(32)	91.13(13)
Ag(3)-Ag(2)-Ag(5)	70.86(11)	S(13)-Ag(25)-Ag(33)	122.1(3)

Ag(11)-Ag(2)-Ag(5)	114.77(12)	S(28)-Ag(25)-Ag(33)	51.2(3)
Ag(1)-Ag(2)-Ag(5)	58.98(10)	Ag(28)-Ag(25)-Ag(33)	74.41(13)
Ag(8)-Ag(2)-Ag(5)	58.32(10)	Ag(26)-Ag(25)-Ag(33)	123.72(14)
Ag(10)-Ag(2)-Ag(5)	128.26(12)	Ag(24)-Ag(25)-Ag(33)	65.43(11)
Ag(15)-Ag(2)-Ag(5)	171.93(12)	Ag(32)-Ag(25)-Ag(33)	131.98(15)
Ag(7)-Ag(2)-Ag(17)	74.43(12)	S(13)-Ag(25)-Ag(31)	49.3(3)
Ag(9)-Ag(2)-Ag(17)	114.30(13)	S(28)-Ag(25)-Ag(31)	134.3(3)
Ag(6)-Ag(2)-Ag(17)	114.80(12)	Ag(28)-Ag(25)-Ag(31)	125.72(15)
Ag(3)-Ag(2)-Ag(17)	57.40(10)	Ag(26)-Ag(25)-Ag(31)	60.95(12)
Ag(11)-Ag(2)-Ag(17)	57.30(10)	Ag(24)-Ag(25)-Ag(31)	67.53(11)
Ag(1)-Ag(2)-Ag(17)	114.23(12)	Ag(32)-Ag(25)-Ag(31)	116.51(15)
Ag(8)-Ag(2)-Ag(17)	131.92(14)	Ag(33)-Ag(25)-Ag(31)	93.39(13)
Ag(10)-Ag(2)-Ag(17)	57.79(10)	S(12)-Ag(26)-S(19)	114.6(4)
Ag(15)-Ag(2)-Ag(17)	85.30(11)	S(12)-Ag(26)-Ag(34)	120.1(3)
Ag(5)-Ag(2)-Ag(17)	96.09(12)	S(19)-Ag(26)-Ag(34)	103.5(3)
S(14)-Ag(3)-S(23)	116.2(4)	S(12)-Ag(26)-Ag(32)	76.1(3)
S(14)-Ag(3)-Ag(1)	119.8(3)	S(19)-Ag(26)-Ag(32)	168.2(3)
S(23)-Ag(3)-Ag(1)	102.8(3)	Ag(34)-Ag(26)-Ag(32)	65.57(13)
S(14)-Ag(3)-Ag(10)	105.3(3)	S(12)-Ag(26)-Ag(25)	106.3(3)
S(23)-Ag(3)-Ag(10)	113.7(3)	S(19)-Ag(26)-Ag(25)	113.9(3)
Ag(1)-Ag(3)-Ag(10)	97.90(15)	Ag(34)-Ag(26)-Ag(25)	97.57(15)
S(14)-Ag(3)-Ag(4)	75.8(3)	Ag(32)-Ag(26)-Ag(25)	65.29(13)
S(23)-Ag(3)-Ag(4)	167.1(3)	S(12)-Ag(26)-Ag(24)	169.9(4)
Ag(1)-Ag(3)-Ag(4)	65.45(13)	S(19)-Ag(26)-Ag(24)	71.2(3)
Ag(10)-Ag(3)-Ag(4)	64.93(12)	Ag(34)-Ag(26)-Ag(24)	64.28(12)
S(14)-Ag(3)-Ag(2)	169.3(3)	Ag(32)-Ag(26)-Ag(24)	98.99(14)
S(23)-Ag(3)-Ag(2)	70.9(2)	Ag(25)-Ag(26)-Ag(24)	63.55(12)
Ag(1)-Ag(3)-Ag(2)	63.64(11)	S(12)-Ag(26)-Ag(31)	105.0(3)
Ag(10)-Ag(3)-Ag(2)	64.03(11)	S(19)-Ag(26)-Ag(31)	56.1(3)
Ag(4)-Ag(3)-Ag(2)	98.10(14)	Ag(34)-Ag(26)-Ag(31)	134.77(16)
S(14)-Ag(3)-Ag(17)	106.4(3)	Ag(32)-Ag(26)-Ag(31)	127.94(17)
S(23)-Ag(3)-Ag(17)	56.2(3)	Ag(25)-Ag(26)-Ag(31)	64.70(12)
Ag(1)-Ag(3)-Ag(17)	133.55(14)	Ag(24)-Ag(26)-Ag(31)	70.69(12)
Ag(10)-Ag(3)-Ag(17)	64.01(11)	S(12)-Ag(26)-Ag(45)	51.3(3)
Ag(4)-Ag(3)-Ag(17)	127.41(15)	S(19)-Ag(26)-Ag(45)	108.2(3)
Ag(2)-Ag(3)-Ag(17)	70.09(11)	Ag(34)-Ag(26)-Ag(45)	74.20(12)
S(14)-Ag(3)-Ag(16)	51.1(3)	Ag(32)-Ag(26)-Ag(45)	74.00(13)
S(23)-Ag(3)-Ag(16)	108.8(3)	Ag(25)-Ag(26)-Ag(45)	137.85(14)
Ag(1)-Ag(3)-Ag(16)	74.43(12)	Ag(24)-Ag(26)-Ag(45)	136.46(14)
Ag(10)-Ag(3)-Ag(16)	137.38(14)	Ag(31)-Ag(26)-Ag(45)	146.77(15)
Ag(4)-Ag(3)-Ag(16)	74.13(12)	S(36)-Ag(27)-S(19)	112.6(4)
Ag(2)-Ag(3)-Ag(16)	136.24(15)	S(36)-Ag(27)-Ag(30)	123.5(3)
Ag(17)-Ag(3)-Ag(16)	147.89(16)	S(19)-Ag(27)-Ag(30)	98.2(3)
S(8)-Ag(4)-S(31)	125.7(5)	S(36)-Ag(27)-Ag(39)	108.2(3)
S(8)-Ag(4)-Ag(9)	121.0(3)	S(19)-Ag(27)-Ag(39)	111.8(3)

S(31)-Ag(4)-Ag(9)	100.9(3)	Ag(30)-Ag(27)-Ag(39)	101.82(16)
S(8)-Ag(4)-Ag(3)	99.9(3)	S(36)-Ag(27)-Ag(42)	74.1(3)
S(31)-Ag(4)-Ag(3)	120.0(4)	S(19)-Ag(27)-Ag(42)	169.5(3)
Ag(9)-Ag(4)-Ag(3)	82.12(13)	Ag(30)-Ag(27)-Ag(42)	71.32(16)
S(8)-Ag(4)-Ag(10)	157.1(3)	Ag(39)-Ag(27)-Ag(42)	72.04(15)
S(31)-Ag(4)-Ag(10)	74.2(4)	S(36)-Ag(27)-Ag(24)	170.7(3)
Ag(9)-Ag(4)-Ag(10)	57.21(11)	S(19)-Ag(27)-Ag(24)	70.1(2)
Ag(3)-Ag(4)-Ag(10)	57.53(11)	Ag(30)-Ag(27)-Ag(24)	63.47(11)
S(8)-Ag(4)-Ag(1)	74.4(3)	Ag(39)-Ag(27)-Ag(24)	63.07(12)
S(31)-Ag(4)-Ag(1)	158.1(3)	Ag(42)-Ag(27)-Ag(24)	104.59(16)
Ag(9)-Ag(4)-Ag(1)	57.79(11)	S(36)-Ag(27)-Ag(43)	106.5(3)
Ag(3)-Ag(4)-Ag(1)	56.66(12)	S(19)-Ag(27)-Ag(43)	54.1(3)
Ag(10)-Ag(4)-Ag(1)	88.46(12)	Ag(30)-Ag(27)-Ag(43)	129.73(15)
S(8)-Ag(4)-Ag(22)	54.6(3)	Ag(39)-Ag(27)-Ag(43)	63.36(13)
S(31)-Ag(4)-Ag(22)	114.0(4)	Ag(42)-Ag(27)-Ag(43)	133.26(18)
Ag(9)-Ag(4)-Ag(22)	75.67(12)	Ag(24)-Ag(27)-Ag(43)	67.45(11)
Ag(3)-Ag(4)-Ag(22)	124.39(16)	S(36)-Ag(27)-Ag(36)	51.4(3)
Ag(10)-Ag(4)-Ag(22)	132.66(14)	S(19)-Ag(27)-Ag(36)	106.1(3)
Ag(1)-Ag(4)-Ag(22)	68.40(12)	Ag(30)-Ag(27)-Ag(36)	75.62(12)
S(8)-Ag(4)-Ag(20)	114.0(3)	Ag(39)-Ag(27)-Ag(36)	141.91(17)
S(31)-Ag(4)-Ag(20)	52.5(3)	Ag(42)-Ag(27)-Ag(36)	71.26(13)
Ag(9)-Ag(4)-Ag(20)	123.27(16)	Ag(24)-Ag(27)-Ag(36)	137.32(15)
Ag(3)-Ag(4)-Ag(20)	75.76(13)	Ag(43)-Ag(27)-Ag(36)	145.93(16)
Ag(10)-Ag(4)-Ag(20)	66.85(12)	S(11)-Ag(28)-S(25)	115.8(4)
Ag(1)-Ag(4)-Ag(20)	132.28(15)	S(11)-Ag(28)-Ag(25)	118.9(3)
Ag(22)-Ag(4)-Ag(20)	156.25(15)	S(25)-Ag(28)-Ag(25)	102.9(3)
S(3)-Ag(5)-S(27)	133.4(5)	S(11)-Ag(28)-Ag(32)	74.8(3)
S(3)-Ag(5)-S(23)	129.5(4)	S(25)-Ag(28)-Ag(32)	168.0(3)
S(27)-Ag(5)-S(23)	95.8(4)	Ag(25)-Ag(28)-Ag(32)	65.86(13)
S(3)-Ag(5)-Ag(7)	78.6(3)	S(11)-Ag(28)-Ag(34)	105.5(3)
S(27)-Ag(5)-Ag(7)	147.2(3)	S(25)-Ag(28)-Ag(34)	114.4(3)
S(23)-Ag(5)-Ag(7)	55.7(2)	Ag(25)-Ag(28)-Ag(34)	98.38(15)
S(3)-Ag(5)-Ag(8)	53.0(3)	Ag(32)-Ag(28)-Ag(34)	65.31(13)
S(27)-Ag(5)-Ag(8)	133.2(4)	S(11)-Ag(28)-Ag(24)	169.5(4)
S(23)-Ag(5)-Ag(8)	104.9(2)	S(25)-Ag(28)-Ag(24)	71.5(3)
Ag(7)-Ag(5)-Ag(8)	54.49(12)	Ag(25)-Ag(28)-Ag(24)	64.09(11)
S(3)-Ag(5)-Ag(1)	122.6(4)	Ag(32)-Ag(28)-Ag(24)	98.76(14)
S(27)-Ag(5)-Ag(1)	53.1(3)	Ag(34)-Ag(28)-Ag(24)	64.04(11)
S(23)-Ag(5)-Ag(1)	93.3(3)	S(11)-Ag(28)-Ag(41)	106.3(3)
Ag(7)-Ag(5)-Ag(1)	107.57(13)	S(25)-Ag(28)-Ag(41)	57.3(3)
Ag(8)-Ag(5)-Ag(1)	83.74(13)	Ag(25)-Ag(28)-Ag(41)	134.67(15)
S(3)-Ag(5)-Ag(2)	107.5(3)	Ag(32)-Ag(28)-Ag(41)	127.19(16)
S(27)-Ag(5)-Ag(2)	102.2(3)	Ag(34)-Ag(28)-Ag(41)	63.77(12)
S(23)-Ag(5)-Ag(2)	62.9(2)	Ag(24)-Ag(28)-Ag(41)	70.76(11)
Ag(7)-Ag(5)-Ag(2)	52.66(9)	S(11)-Ag(28)-Ag(29)	50.4(3)

Ag(8)-Ag(5)-Ag(2)	55.23(10)	S(25)-Ag(28)-Ag(29)	108.2(3)
Ag(1)-Ag(5)-Ag(2)	54.94(9)	Ag(25)-Ag(28)-Ag(29)	74.07(12)
S(22)-Ag(6)-S(26)	111.6(3)	Ag(32)-Ag(28)-Ag(29)	73.65(12)
S(22)-Ag(6)-Ag(8)	122.7(3)	Ag(34)-Ag(28)-Ag(29)	137.34(15)
S(26)-Ag(6)-Ag(8)	97.6(2)	Ag(24)-Ag(28)-Ag(29)	136.38(14)
S(22)-Ag(6)-Ag(11)	108.6(3)	Ag(41)-Ag(28)-Ag(29)	147.45(15)
S(26)-Ag(6)-Ag(11)	112.0(3)	P(10)-Ag(29)-S(33)	127.7(5)
Ag(8)-Ag(6)-Ag(11)	103.75(14)	P(10)-Ag(29)-S(28)	115.7(5)
S(22)-Ag(6)-Ag(18)	73.3(3)	S(33)-Ag(29)-S(28)	96.6(4)
S(26)-Ag(6)-Ag(18)	171.8(3)	P(10)-Ag(29)-S(11)	109.2(5)
Ag(8)-Ag(6)-Ag(18)	74.24(14)	S(33)-Ag(29)-S(11)	93.9(4)
Ag(11)-Ag(6)-Ag(18)	71.20(14)	S(28)-Ag(29)-S(11)	111.4(4)
S(22)-Ag(6)-Ag(2)	171.4(3)	P(10)-Ag(29)-Ag(28)	144.1(5)
S(26)-Ag(6)-Ag(2)	70.7(2)	S(33)-Ag(29)-Ag(28)	85.4(3)
Ag(8)-Ag(6)-Ag(2)	64.20(11)	S(28)-Ag(29)-Ag(28)	66.1(3)
Ag(11)-Ag(6)-Ag(2)	63.28(10)	S(11)-Ag(29)-Ag(28)	47.5(3)
Ag(18)-Ag(6)-Ag(2)	105.43(14)	S(15)-Ag(30)-S(7)	120.8(4)
S(22)-Ag(6)-Ag(15)	107.9(3)	S(15)-Ag(30)-Ag(27)	157.0(3)
S(26)-Ag(6)-Ag(15)	53.4(2)	S(7)-Ag(30)-Ag(27)	79.2(3)
Ag(8)-Ag(6)-Ag(15)	128.83(13)	S(15)-Ag(30)-Ag(38)	81.1(3)
Ag(11)-Ag(6)-Ag(15)	63.31(11)	S(7)-Ag(30)-Ag(38)	156.1(3)
Ag(18)-Ag(6)-Ag(15)	132.36(16)	Ag(27)-Ag(30)-Ag(38)	77.53(14)
Ag(2)-Ag(6)-Ag(15)	66.31(10)	S(15)-Ag(30)-Ag(24)	115.1(3)
S(22)-Ag(6)-Ag(12)	51.7(3)	S(7)-Ag(30)-Ag(24)	112.5(3)
S(26)-Ag(6)-Ag(12)	105.2(2)	Ag(27)-Ag(30)-Ag(24)	59.94(11)
Ag(8)-Ag(6)-Ag(12)	73.93(12)	Ag(38)-Ag(30)-Ag(24)	59.14(11)
Ag(11)-Ag(6)-Ag(12)	142.59(14)	S(15)-Ag(30)-Ag(31)	120.6(3)
Ag(18)-Ag(6)-Ag(12)	72.38(14)	S(7)-Ag(30)-Ag(31)	51.5(3)
Ag(2)-Ag(6)-Ag(12)	136.51(14)	Ag(27)-Ag(30)-Ag(31)	80.10(14)
Ag(15)-Ag(6)-Ag(12)	146.61(14)	Ag(38)-Ag(30)-Ag(31)	128.24(14)
S(2)-Ag(7)-S(23)	111.0(4)	Ag(24)-Ag(30)-Ag(31)	69.21(11)
S(2)-Ag(7)-Ag(11)	125.6(3)	S(15)-Ag(30)-Ag(33)	50.1(3)
S(23)-Ag(7)-Ag(11)	97.6(3)	S(7)-Ag(30)-Ag(33)	138.8(3)
S(2)-Ag(7)-Ag(8)	106.7(4)	Ag(27)-Ag(30)-Ag(33)	123.37(14)
S(23)-Ag(7)-Ag(8)	112.6(3)	Ag(38)-Ag(30)-Ag(33)	61.57(12)
Ag(11)-Ag(7)-Ag(8)	102.96(15)	Ag(24)-Ag(30)-Ag(33)	65.73(10)
S(2)-Ag(7)-Ag(18)	74.4(3)	Ag(31)-Ag(30)-Ag(33)	95.30(13)
S(23)-Ag(7)-Ag(18)	168.8(3)	S(15)-Ag(30)-Ag(42)	105.1(3)
Ag(11)-Ag(7)-Ag(18)	71.62(14)	S(7)-Ag(30)-Ag(42)	105.8(3)
Ag(8)-Ag(7)-Ag(18)	73.82(15)	Ag(27)-Ag(30)-Ag(42)	55.09(13)
S(2)-Ag(7)-Ag(2)	169.3(4)	Ag(38)-Ag(30)-Ag(42)	55.46(12)
S(23)-Ag(7)-Ag(2)	70.3(2)	Ag(24)-Ag(30)-Ag(42)	92.52(13)
Ag(11)-Ag(7)-Ag(2)	63.70(11)	Ag(31)-Ag(30)-Ag(42)	134.31(15)
Ag(8)-Ag(7)-Ag(2)	63.92(12)	Ag(33)-Ag(30)-Ag(42)	115.36(14)
Ag(18)-Ag(7)-Ag(2)	106.38(15)	S(13)-Ag(31)-S(7)	139.8(4)

S(2)-Ag(7)-Ag(5)	102.5(3)	S(13)-Ag(31)-S(19)	126.6(4)
S(23)-Ag(7)-Ag(5)	54.9(3)	S(7)-Ag(31)-S(19)	92.6(4)
Ag(11)-Ag(7)-Ag(5)	131.49(14)	S(13)-Ag(31)-Ag(26)	77.1(3)
Ag(8)-Ag(7)-Ag(5)	63.86(12)	S(7)-Ag(31)-Ag(26)	142.6(3)
Ag(18)-Ag(7)-Ag(5)	134.81(18)	S(19)-Ag(31)-Ag(26)	53.3(2)
Ag(2)-Ag(7)-Ag(5)	69.11(11)	S(13)-Ag(31)-Ag(30)	124.4(3)
S(2)-Ag(7)-Ag(13)	52.3(3)	S(7)-Ag(31)-Ag(30)	53.6(3)
S(23)-Ag(7)-Ag(13)	104.6(3)	S(19)-Ag(31)-Ag(30)	91.0(3)
Ag(11)-Ag(7)-Ag(13)	76.60(12)	Ag(26)-Ag(31)-Ag(30)	106.25(14)
Ag(8)-Ag(7)-Ag(13)	142.39(15)	S(13)-Ag(31)-Ag(25)	52.1(3)
Ag(18)-Ag(7)-Ag(13)	70.45(13)	S(7)-Ag(31)-Ag(25)	135.6(3)
Ag(2)-Ag(7)-Ag(13)	138.27(15)	S(19)-Ag(31)-Ag(25)	102.2(2)
Ag(5)-Ag(7)-Ag(13)	142.97(15)	Ag(26)-Ag(31)-Ag(25)	54.36(11)
S(20)-Ag(8)-S(3)	120.7(5)	Ag(30)-Ag(31)-Ag(25)	84.18(13)
S(20)-Ag(8)-Ag(6)	80.4(3)	S(33)-Ag(32)-S(1)	126.3(5)
S(3)-Ag(8)-Ag(6)	155.5(4)	S(33)-Ag(32)-Ag(26)	119.7(3)
S(20)-Ag(8)-Ag(7)	155.9(3)	S(1)-Ag(32)-Ag(26)	100.6(3)
S(3)-Ag(8)-Ag(7)	81.6(4)	S(33)-Ag(32)-Ag(28)	100.4(4)
Ag(6)-Ag(8)-Ag(7)	75.94(13)	S(1)-Ag(32)-Ag(28)	120.4(4)
S(20)-Ag(8)-Ag(2)	112.2(3)	Ag(26)-Ag(32)-Ag(28)	81.75(14)
S(3)-Ag(8)-Ag(2)	115.9(3)	S(33)-Ag(32)-Ag(25)	73.8(3)
Ag(6)-Ag(8)-Ag(2)	59.44(10)	S(1)-Ag(32)-Ag(25)	157.7(3)
Ag(7)-Ag(8)-Ag(2)	58.54(11)	Ag(26)-Ag(32)-Ag(25)	57.56(12)
S(20)-Ag(8)-Ag(19)	51.3(3)	Ag(28)-Ag(32)-Ag(25)	56.41(12)
S(3)-Ag(8)-Ag(19)	121.1(3)	S(33)-Ag(32)-Ag(34)	157.3(4)
Ag(6)-Ag(8)-Ag(19)	81.19(13)	S(1)-Ag(32)-Ag(34)	74.2(3)
Ag(7)-Ag(8)-Ag(19)	127.67(15)	Ag(26)-Ag(32)-Ag(34)	57.17(11)
Ag(2)-Ag(8)-Ag(19)	69.24(11)	Ag(28)-Ag(32)-Ag(34)	57.44(12)
S(20)-Ag(8)-Ag(5)	138.7(3)	Ag(25)-Ag(32)-Ag(34)	88.30(13)
S(3)-Ag(8)-Ag(5)	50.2(3)	S(33)-Ag(32)-Ag(44)	53.3(3)
Ag(6)-Ag(8)-Ag(5)	123.16(15)	S(1)-Ag(32)-Ag(44)	115.4(4)
Ag(7)-Ag(8)-Ag(5)	61.65(12)	Ag(26)-Ag(32)-Ag(44)	74.62(12)
Ag(2)-Ag(8)-Ag(5)	66.46(11)	Ag(28)-Ag(32)-Ag(44)	122.21(17)
Ag(19)-Ag(8)-Ag(5)	95.64(13)	Ag(25)-Ag(32)-Ag(44)	66.41(13)
S(20)-Ag(8)-Ag(18)	107.9(3)	Ag(34)-Ag(32)-Ag(44)	131.69(14)
S(3)-Ag(8)-Ag(18)	104.2(3)	S(33)-Ag(32)-Ag(40)	114.8(3)
Ag(6)-Ag(8)-Ag(18)	54.06(11)	S(1)-Ag(32)-Ag(40)	52.9(3)
Ag(7)-Ag(8)-Ag(18)	53.47(12)	Ag(26)-Ag(32)-Ag(40)	123.93(16)
Ag(2)-Ag(8)-Ag(18)	90.62(14)	Ag(28)-Ag(32)-Ag(40)	76.45(12)
Ag(19)-Ag(8)-Ag(18)	134.71(14)	Ag(25)-Ag(32)-Ag(40)	132.69(15)
Ag(5)-Ag(8)-Ag(18)	113.34(15)	Ag(34)-Ag(32)-Ag(40)	67.57(12)
S(17)-Ag(9)-S(26)	113.6(4)	Ag(44)-Ag(32)-Ag(40)	157.44(16)
S(17)-Ag(9)-Ag(10)	117.8(3)	S(15)-Ag(33)-S(28)	132.6(4)
S(26)-Ag(9)-Ag(10)	105.1(2)	S(15)-Ag(33)-S(25)	129.5(4)
S(17)-Ag(9)-Ag(4)	76.0(3)	S(28)-Ag(33)-S(25)	96.2(4)

S(26)-Ag(9)-Ag(4)	169.4(3)	S(15)-Ag(33)-Ag(38)	78.2(3)
Ag(10)-Ag(9)-Ag(4)	65.35(12)	S(28)-Ag(33)-Ag(38)	148.5(3)
S(17)-Ag(9)-Ag(1)	108.4(3)	S(25)-Ag(33)-Ag(38)	56.6(3)
S(26)-Ag(9)-Ag(1)	113.7(3)	S(15)-Ag(33)-Ag(25)	122.4(3)
Ag(10)-Ag(9)-Ag(1)	97.31(14)	S(28)-Ag(33)-Ag(25)	52.8(3)
Ag(4)-Ag(9)-Ag(1)	65.03(12)	S(25)-Ag(33)-Ag(25)	95.1(3)
S(17)-Ag(9)-Ag(2)	171.7(3)	Ag(38)-Ag(33)-Ag(25)	109.34(14)
S(26)-Ag(9)-Ag(2)	72.2(2)	S(15)-Ag(33)-Ag(30)	52.8(3)
Ag(10)-Ag(9)-Ag(2)	64.49(11)	S(28)-Ag(33)-Ag(30)	133.5(3)
Ag(4)-Ag(9)-Ag(2)	98.81(13)	S(25)-Ag(33)-Ag(30)	106.1(3)
Ag(1)-Ag(9)-Ag(2)	63.31(11)	Ag(38)-Ag(33)-Ag(30)	54.66(11)
S(17)-Ag(9)-Ag(19)	106.3(3)	Ag(25)-Ag(33)-Ag(30)	84.46(12)
S(26)-Ag(9)-Ag(19)	56.2(2)	S(15)-Ag(33)-Ag(24)	107.4(3)
Ag(10)-Ag(9)-Ag(19)	135.83(15)	S(28)-Ag(33)-Ag(24)	102.9(3)
Ag(4)-Ag(9)-Ag(19)	126.98(15)	S(25)-Ag(33)-Ag(24)	64.6(3)
Ag(1)-Ag(9)-Ag(19)	64.32(12)	Ag(38)-Ag(33)-Ag(24)	53.49(10)
Ag(2)-Ag(9)-Ag(19)	71.46(12)	Ag(25)-Ag(33)-Ag(24)	55.87(10)
S(10)-Ag(10)-S(29)	120.7(4)	Ag(30)-Ag(33)-Ag(24)	55.25(9)
S(10)-Ag(10)-Ag(9)	161.1(3)	S(21)-Ag(34)-S(30)	121.0(4)
S(29)-Ag(10)-Ag(9)	77.1(3)	S(21)-Ag(34)-Ag(26)	159.8(3)
S(10)-Ag(10)-Ag(3)	79.8(3)	S(30)-Ag(34)-Ag(26)	77.2(3)
S(29)-Ag(10)-Ag(3)	159.3(3)	S(21)-Ag(34)-Ag(28)	79.8(3)
Ag(9)-Ag(10)-Ag(3)	82.25(13)	S(30)-Ag(34)-Ag(28)	158.9(3)
S(10)-Ag(10)-Ag(2)	115.4(3)	Ag(26)-Ag(34)-Ag(28)	81.70(13)
S(29)-Ag(10)-Ag(2)	109.6(3)	S(21)-Ag(34)-Ag(24)	116.6(3)
Ag(9)-Ag(10)-Ag(2)	58.36(10)	S(30)-Ag(34)-Ag(24)	109.2(3)
Ag(3)-Ag(10)-Ag(2)	58.57(10)	Ag(26)-Ag(34)-Ag(24)	58.50(11)
S(10)-Ag(10)-Ag(4)	107.1(3)	Ag(28)-Ag(34)-Ag(24)	58.47(11)
S(29)-Ag(10)-Ag(4)	109.1(3)	S(21)-Ag(34)-Ag(32)	105.3(3)
Ag(9)-Ag(10)-Ag(4)	57.44(11)	S(30)-Ag(34)-Ag(32)	109.4(3)
Ag(3)-Ag(10)-Ag(4)	57.54(11)	Ag(26)-Ag(34)-Ag(32)	57.27(11)
Ag(2)-Ag(10)-Ag(4)	90.51(12)	Ag(28)-Ag(34)-Ag(32)	57.25(12)
S(10)-Ag(10)-Ag(15)	123.0(3)	Ag(24)-Ag(34)-Ag(32)	90.64(13)
S(29)-Ag(10)-Ag(15)	51.1(3)	S(21)-Ag(34)-Ag(41)	50.0(3)
Ag(9)-Ag(10)-Ag(15)	72.22(11)	S(30)-Ag(34)-Ag(41)	133.3(3)
Ag(3)-Ag(10)-Ag(15)	122.49(14)	Ag(26)-Ag(34)-Ag(41)	125.37(15)
Ag(2)-Ag(10)-Ag(15)	64.15(10)	Ag(28)-Ag(34)-Ag(41)	61.34(12)
Ag(4)-Ag(10)-Ag(15)	129.52(14)	Ag(24)-Ag(34)-Ag(41)	67.85(11)
S(10)-Ag(10)-Ag(17)	49.3(3)	Ag(32)-Ag(34)-Ag(41)	117.07(15)
S(29)-Ag(10)-Ag(17)	133.1(3)	S(21)-Ag(34)-Ag(43)	123.7(3)
Ag(9)-Ag(10)-Ag(17)	124.76(14)	S(30)-Ag(34)-Ag(43)	50.6(3)
Ag(3)-Ag(10)-Ag(17)	61.32(11)	Ag(26)-Ag(34)-Ag(43)	73.66(12)
Ag(2)-Ag(10)-Ag(17)	67.18(11)	Ag(28)-Ag(34)-Ag(43)	122.96(14)
Ag(4)-Ag(10)-Ag(17)	117.61(13)	Ag(24)-Ag(34)-Ag(43)	64.77(11)
Ag(15)-Ag(10)-Ag(17)	92.93(12)	Ag(32)-Ag(34)-Ag(43)	130.76(14)

S(16)-Ag(11)-S(18)	124.2(4)	Ag(41)-Ag(34)-Ag(43)	93.29(13)
S(16)-Ag(11)-Ag(7)	156.1(3)	P(12)-Ag(35)-S(35)	120.0(6)
S(18)-Ag(11)-Ag(7)	77.6(3)	P(12)-Ag(35)-S(4)	107.0(5)
S(16)-Ag(11)-Ag(6)	81.8(3)	S(35)-Ag(35)-S(4)	100.6(5)
S(18)-Ag(11)-Ag(6)	152.5(3)	P(12)-Ag(35)-S(9)	116.0(5)
Ag(7)-Ag(11)-Ag(6)	75.39(13)	S(35)-Ag(35)-S(9)	98.2(5)
S(16)-Ag(11)-Ag(2)	114.4(3)	S(4)-Ag(35)-S(9)	114.2(4)
S(18)-Ag(11)-Ag(2)	109.7(3)	P(12)-Ag(35)-Ag(38)	152.7(5)
Ag(7)-Ag(11)-Ag(2)	58.84(10)	S(35)-Ag(35)-Ag(38)	87.0(4)
Ag(6)-Ag(11)-Ag(2)	59.16(10)	S(4)-Ag(35)-Ag(38)	68.7(3)
S(16)-Ag(11)-Ag(17)	120.0(3)	S(9)-Ag(35)-Ag(38)	50.2(3)
S(18)-Ag(11)-Ag(17)	51.1(3)	P(11)-Ag(36)-S(36)	113.0(5)
Ag(7)-Ag(11)-Ag(17)	80.43(13)	P(11)-Ag(36)-S(6)	121.3(5)
Ag(6)-Ag(11)-Ag(17)	127.42(13)	S(36)-Ag(36)-S(6)	102.1(5)
Ag(2)-Ag(11)-Ag(17)	68.33(11)	P(11)-Ag(36)-S(7)	108.3(5)
S(16)-Ag(11)-Ag(15)	50.5(3)	S(36)-Ag(36)-S(7)	116.1(4)
S(18)-Ag(11)-Ag(15)	139.2(3)	S(6)-Ag(36)-S(7)	95.2(4)
Ag(7)-Ag(11)-Ag(15)	121.19(14)	P(11)-Ag(36)-Ag(27)	150.0(4)
Ag(6)-Ag(11)-Ag(15)	62.50(11)	S(36)-Ag(36)-Ag(27)	50.2(3)
Ag(2)-Ag(11)-Ag(15)	64.70(10)	S(6)-Ag(36)-Ag(27)	88.4(3)
Ag(17)-Ag(11)-Ag(15)	93.75(12)	S(7)-Ag(36)-Ag(27)	69.7(2)
S(16)-Ag(11)-Ag(18)	105.9(3)	P(13)-Ag(37)-S(9)	121.9(4)
S(18)-Ag(11)-Ag(18)	104.4(3)	P(13)-Ag(37)-S(15)	117.6(4)
Ag(7)-Ag(11)-Ag(18)	54.75(11)	S(9)-Ag(37)-S(15)	93.6(4)
Ag(6)-Ag(11)-Ag(18)	54.99(11)	P(13)-Ag(37)-S(6)	107.5(4)
Ag(2)-Ag(11)-Ag(18)	92.73(13)	S(9)-Ag(37)-S(6)	108.9(5)
Ag(17)-Ag(11)-Ag(18)	134.17(14)	S(15)-Ag(37)-S(6)	106.1(4)
Ag(15)-Ag(11)-Ag(18)	115.99(13)	S(9)-Ag(38)-S(25)	112.4(4)
P(14)-Ag(12)-S(32)	121.8(5)	S(9)-Ag(38)-Ag(39)	123.5(3)
P(14)-Ag(12)-S(22)	117.4(4)	S(25)-Ag(38)-Ag(39)	99.1(3)
S(32)-Ag(12)-S(22)	97.1(5)	S(9)-Ag(38)-Ag(30)	107.9(3)
P(14)-Ag(12)-S(20)	105.6(4)	S(25)-Ag(38)-Ag(30)	111.7(3)
S(32)-Ag(12)-S(20)	99.7(5)	Ag(39)-Ag(38)-Ag(30)	101.52(15)
S(22)-Ag(12)-S(20)	114.3(3)	S(9)-Ag(38)-Ag(42)	74.2(3)
P(14)-Ag(12)-Ag(6)	152.5(4)	S(25)-Ag(38)-Ag(42)	171.2(3)
S(32)-Ag(12)-Ag(6)	85.6(4)	Ag(39)-Ag(38)-Ag(42)	72.06(15)
S(22)-Ag(12)-Ag(6)	49.3(2)	Ag(30)-Ag(38)-Ag(42)	70.17(15)
S(20)-Ag(12)-Ag(6)	69.3(3)	S(9)-Ag(38)-Ag(24)	170.4(3)
P(2)-Ag(13)-S(2)	110.9(7)	S(25)-Ag(38)-Ag(24)	70.4(3)
P(2)-Ag(13)-S(5)	118.6(5)	Ag(39)-Ag(38)-Ag(24)	63.67(12)
S(2)-Ag(13)-S(5)	104.9(5)	Ag(30)-Ag(38)-Ag(24)	63.07(11)
P(2)-Ag(13)-S(18)	111.1(6)	Ag(42)-Ag(38)-Ag(24)	104.09(16)
S(2)-Ag(13)-S(18)	116.2(4)	S(9)-Ag(38)-Ag(33)	105.9(3)
S(5)-Ag(13)-S(18)	94.5(4)	S(25)-Ag(38)-Ag(33)	53.6(3)
P(2)-Ag(13)-Ag(7)	151.1(4)	Ag(39)-Ag(38)-Ag(33)	130.38(15)

S(2)-Ag(13)-Ag(7)	51.0(3)	Ag(30)-Ag(38)-Ag(33)	63.77(11)
S(5)-Ag(13)-Ag(7)	89.7(3)	Ag(42)-Ag(38)-Ag(33)	131.55(17)
S(18)-Ag(13)-Ag(7)	69.4(3)	Ag(24)-Ag(38)-Ag(33)	67.85(11)
P(3)-Ag(14)-S(16)	115.6(4)	S(9)-Ag(38)-Ag(35)	52.9(3)
P(3)-Ag(14)-S(5)	106.9(4)	S(25)-Ag(38)-Ag(35)	106.7(3)
S(16)-Ag(14)-S(5)	108.8(4)	Ag(39)-Ag(38)-Ag(35)	74.05(13)
P(3)-Ag(14)-S(22)	121.0(4)	Ag(30)-Ag(38)-Ag(35)	141.49(15)
S(16)-Ag(14)-S(22)	95.1(4)	Ag(42)-Ag(38)-Ag(35)	72.21(14)
S(5)-Ag(14)-S(22)	108.6(4)	Ag(24)-Ag(38)-Ag(35)	136.01(15)
P(3)-Ag(14)-Ag(18)	141.2(3)	Ag(33)-Ag(38)-Ag(35)	146.87(16)
S(16)-Ag(14)-Ag(18)	101.5(3)	S(34)-Ag(39)-S(4)	122.4(4)
S(5)-Ag(14)-Ag(18)	46.8(3)	S(34)-Ag(39)-Ag(38)	157.1(4)
S(22)-Ag(14)-Ag(18)	63.2(2)	S(4)-Ag(39)-Ag(38)	77.8(3)
S(16)-Ag(15)-S(29)	130.4(4)	S(34)-Ag(39)-Ag(27)	81.4(3)
S(16)-Ag(15)-S(26)	127.7(4)	S(4)-Ag(39)-Ag(27)	155.0(4)
S(29)-Ag(15)-S(26)	99.4(4)	Ag(38)-Ag(39)-Ag(27)	77.45(14)
S(16)-Ag(15)-Ag(6)	77.9(3)	S(34)-Ag(39)-Ag(24)	115.8(3)
S(29)-Ag(15)-Ag(6)	151.2(3)	S(4)-Ag(39)-Ag(24)	109.5(3)
S(26)-Ag(15)-Ag(6)	56.2(2)	Ag(38)-Ag(39)-Ag(24)	59.48(11)
S(16)-Ag(15)-Ag(10)	123.0(3)	Ag(27)-Ag(39)-Ag(24)	59.43(11)
S(29)-Ag(15)-Ag(10)	53.1(3)	S(34)-Ag(39)-Ag(43)	50.8(3)
S(26)-Ag(15)-Ag(10)	97.6(2)	S(4)-Ag(39)-Ag(43)	137.2(3)
Ag(6)-Ag(15)-Ag(10)	110.49(12)	Ag(38)-Ag(39)-Ag(43)	123.17(15)
S(16)-Ag(15)-Ag(11)	53.0(3)	Ag(27)-Ag(39)-Ag(43)	62.17(12)
S(29)-Ag(15)-Ag(11)	134.0(3)	Ag(24)-Ag(39)-Ag(43)	65.81(11)
S(26)-Ag(15)-Ag(11)	106.2(3)	S(34)-Ag(39)-Ag(41)	120.2(4)
Ag(6)-Ag(15)-Ag(11)	54.19(10)	S(4)-Ag(39)-Ag(41)	50.6(3)
Ag(10)-Ag(15)-Ag(11)	85.61(11)	Ag(38)-Ag(39)-Ag(41)	80.22(14)
S(16)-Ag(15)-Ag(2)	108.2(3)	Ag(27)-Ag(39)-Ag(41)	127.11(15)
S(29)-Ag(15)-Ag(2)	104.7(3)	Ag(24)-Ag(39)-Ag(41)	67.88(11)
S(26)-Ag(15)-Ag(2)	65.6(2)	Ag(43)-Ag(39)-Ag(41)	93.28(14)
Ag(6)-Ag(15)-Ag(2)	53.68(9)	S(34)-Ag(39)-Ag(42)	104.9(3)
Ag(10)-Ag(15)-Ag(2)	56.86(9)	S(4)-Ag(39)-Ag(42)	107.6(3)
Ag(11)-Ag(15)-Ag(2)	55.84(9)	Ag(38)-Ag(39)-Ag(42)	55.05(13)
P(6)-Ag(16)-S(8)	126.9(5)	Ag(27)-Ag(39)-Ag(42)	54.10(13)
P(6)-Ag(16)-S(27)	114.2(5)	Ag(24)-Ag(39)-Ag(42)	91.37(15)
S(8)-Ag(16)-S(27)	97.7(4)	Ag(43)-Ag(39)-Ag(42)	114.85(15)
P(6)-Ag(16)-S(14)	108.8(6)	Ag(41)-Ag(39)-Ag(42)	134.85(16)
S(8)-Ag(16)-S(14)	94.6(4)	P(9)-Ag(40)-S(11)	122.2(5)
S(27)-Ag(16)-S(14)	113.1(4)	P(9)-Ag(40)-S(1)	106.6(5)
P(6)-Ag(16)-Ag(3)	144.2(5)	S(11)-Ag(40)-S(1)	115.5(4)
S(8)-Ag(16)-Ag(3)	86.3(3)	P(9)-Ag(40)-S(21)	116.8(5)
S(27)-Ag(16)-Ag(3)	67.1(3)	S(11)-Ag(40)-S(21)	88.6(4)
S(14)-Ag(16)-Ag(3)	48.4(3)	S(1)-Ag(40)-S(21)	105.3(4)
S(10)-Ag(17)-S(18)	142.6(4)	P(9)-Ag(40)-Ag(32)	145.4(4)

S(10)-Ag(17)-S(23)	126.4(4)	S(11)-Ag(40)-Ag(32)	66.9(3)
S(18)-Ag(17)-S(23)	89.4(4)	S(1)-Ag(40)-Ag(32)	49.5(3)
S(10)-Ag(17)-Ag(3)	77.5(3)	S(21)-Ag(40)-Ag(32)	95.7(3)
S(18)-Ag(17)-Ag(3)	139.9(3)	S(21)-Ag(41)-S(4)	143.6(5)
S(23)-Ag(17)-Ag(3)	53.4(2)	S(21)-Ag(41)-S(25)	125.9(4)
S(10)-Ag(17)-Ag(11)	126.5(3)	S(4)-Ag(41)-S(25)	89.5(4)
S(18)-Ag(17)-Ag(11)	53.3(3)	S(21)-Ag(41)-Ag(28)	76.8(3)
S(23)-Ag(17)-Ag(11)	91.0(2)	S(4)-Ag(41)-Ag(28)	139.3(3)
Ag(3)-Ag(17)-Ag(11)	106.86(13)	S(25)-Ag(41)-Ag(28)	53.4(3)
S(10)-Ag(17)-Ag(10)	52.9(3)	S(21)-Ag(41)-Ag(34)	52.0(3)
S(18)-Ag(17)-Ag(10)	137.4(3)	S(4)-Ag(41)-Ag(34)	135.5(4)
S(23)-Ag(17)-Ag(10)	102.8(3)	S(25)-Ag(41)-Ag(34)	102.9(3)
Ag(3)-Ag(17)-Ag(10)	54.68(10)	Ag(28)-Ag(41)-Ag(34)	54.88(11)
Ag(11)-Ag(17)-Ag(10)	85.43(11)	S(21)-Ag(41)-Ag(39)	126.3(4)
S(10)-Ag(17)-Ag(2)	107.0(3)	S(4)-Ag(41)-Ag(39)	52.0(3)
S(18)-Ag(17)-Ag(2)	99.5(3)	S(25)-Ag(41)-Ag(39)	90.0(2)
S(23)-Ag(17)-Ag(2)	61.6(2)	Ag(28)-Ag(41)-Ag(39)	105.83(13)
Ag(3)-Ag(17)-Ag(2)	52.51(10)	Ag(34)-Ag(41)-Ag(39)	84.97(13)
Ag(11)-Ag(17)-Ag(2)	54.37(9)	S(35)-Ag(42)-S(6)	136.9(6)
Ag(10)-Ag(17)-Ag(2)	55.03(9)	S(35)-Ag(42)-Ag(27)	113.1(5)
S(32)-Ag(18)-S(5)	139.0(5)	S(6)-Ag(42)-Ag(27)	100.8(4)
S(32)-Ag(18)-Ag(7)	111.3(4)	S(35)-Ag(42)-Ag(38)	100.2(4)
S(5)-Ag(18)-Ag(7)	100.8(3)	S(6)-Ag(42)-Ag(38)	113.7(4)
S(32)-Ag(18)-Ag(6)	99.2(4)	Ag(27)-Ag(42)-Ag(38)	76.07(16)
S(5)-Ag(18)-Ag(6)	113.9(4)	S(35)-Ag(42)-Ag(30)	151.7(4)
Ag(7)-Ag(18)-Ag(6)	74.13(15)	S(6)-Ag(42)-Ag(30)	70.8(4)
S(32)-Ag(18)-Ag(11)	150.2(4)	Ag(27)-Ag(42)-Ag(30)	53.59(13)
S(5)-Ag(18)-Ag(11)	70.4(3)	Ag(38)-Ag(42)-Ag(30)	54.37(13)
Ag(7)-Ag(18)-Ag(11)	53.63(12)	S(35)-Ag(42)-Ag(39)	70.9(4)
Ag(6)-Ag(18)-Ag(11)	53.81(12)	S(6)-Ag(42)-Ag(39)	151.6(4)
S(32)-Ag(18)-Ag(14)	126.9(3)	Ag(27)-Ag(42)-Ag(39)	53.86(12)
S(5)-Ag(18)-Ag(14)	51.0(3)	Ag(38)-Ag(42)-Ag(39)	52.89(12)
Ag(7)-Ag(18)-Ag(14)	115.53(19)	Ag(30)-Ag(42)-Ag(39)	82.47(14)
Ag(6)-Ag(18)-Ag(14)	71.76(12)	S(34)-Ag(43)-S(30)	133.7(4)
Ag(11)-Ag(18)-Ag(14)	62.00(11)	S(34)-Ag(43)-S(19)	127.4(4)
S(32)-Ag(18)-Ag(8)	69.8(4)	S(30)-Ag(43)-S(19)	97.2(4)
S(5)-Ag(18)-Ag(8)	150.5(4)	S(34)-Ag(43)-Ag(27)	77.5(3)
Ag(7)-Ag(18)-Ag(8)	52.71(12)	S(30)-Ag(43)-Ag(27)	148.3(3)
Ag(6)-Ag(18)-Ag(8)	51.70(11)	S(19)-Ag(43)-Ag(27)	55.1(3)
Ag(11)-Ag(18)-Ag(8)	81.96(13)	S(34)-Ag(43)-Ag(39)	52.6(3)
Ag(14)-Ag(18)-Ag(8)	123.46(15)	S(30)-Ag(43)-Ag(39)	134.1(3)
S(24)-Ag(19)-S(20)	141.0(4)	S(19)-Ag(43)-Ag(39)	104.7(3)
S(24)-Ag(19)-S(26)	126.5(4)	Ag(27)-Ag(43)-Ag(39)	54.46(12)
S(20)-Ag(19)-S(26)	91.6(4)	S(34)-Ag(43)-Ag(34)	123.9(4)
S(24)-Ag(19)-Ag(9)	77.1(3)	S(30)-Ag(43)-Ag(34)	52.4(3)

S(20)-Ag(19)-Ag(9)	141.3(3)	S(19)-Ag(43)-Ag(34)	94.9(3)
S(26)-Ag(19)-Ag(9)	53.6(2)	Ag(27)-Ag(43)-Ag(34)	109.59(14)
S(24)-Ag(19)-Ag(8)	124.7(4)	Ag(39)-Ag(43)-Ag(34)	85.55(13)
S(20)-Ag(19)-Ag(8)	52.6(3)	S(34)-Ag(43)-Ag(24)	107.2(3)
S(26)-Ag(19)-Ag(8)	90.7(2)	S(30)-Ag(43)-Ag(24)	102.8(3)
Ag(9)-Ag(19)-Ag(8)	105.62(14)	S(19)-Ag(43)-Ag(24)	63.9(3)
S(24)-Ag(19)-Ag(1)	52.3(3)	Ag(27)-Ag(43)-Ag(24)	53.59(10)
S(20)-Ag(19)-Ag(1)	134.4(4)	Ag(39)-Ag(43)-Ag(24)	55.39(10)
S(26)-Ag(19)-Ag(1)	102.7(3)	Ag(34)-Ag(43)-Ag(24)	56.00(10)
Ag(9)-Ag(19)-Ag(1)	54.56(10)	P(7)-Ag(44)-S(12)	119.9(5)
Ag(8)-Ag(19)-Ag(1)	83.70(12)	P(7)-Ag(44)-S(33)	105.5(4)
P(15)-Ag(20)-S(14)	122.2(6)	S(12)-Ag(44)-S(33)	116.8(5)
P(15)-Ag(20)-S(31)	108.2(5)	P(7)-Ag(44)-S(13)	114.9(4)
S(14)-Ag(20)-S(31)	116.2(4)	S(12)-Ag(44)-S(13)	92.0(4)
P(15)-Ag(20)-S(10)	112.4(5)	S(33)-Ag(44)-S(13)	106.9(4)
S(14)-Ag(20)-S(10)	88.5(4)	P(7)-Ag(44)-Ag(32)	144.3(3)
S(31)-Ag(20)-S(10)	107.0(4)	S(12)-Ag(44)-Ag(32)	69.3(3)
P(15)-Ag(20)-Ag(4)	148.7(4)	S(33)-Ag(44)-Ag(32)	49.0(3)
S(14)-Ag(20)-Ag(4)	67.8(3)	S(13)-Ag(44)-Ag(32)	98.2(3)
S(31)-Ag(20)-Ag(4)	49.4(3)	P(8)-Ag(45)-S(1)	129.5(5)
S(10)-Ag(20)-Ag(4)	96.7(3)	P(8)-Ag(45)-S(30)	112.9(5)
P(4)-Ag(21)-S(31)	130.6(5)	S(1)-Ag(45)-S(30)	96.2(4)
P(4)-Ag(21)-S(17)	112.1(5)	P(8)-Ag(45)-S(12)	109.6(5)
S(31)-Ag(21)-S(17)	93.7(4)	S(1)-Ag(45)-S(12)	94.8(5)
P(4)-Ag(21)-S(29)	113.4(4)	S(30)-Ag(45)-S(12)	112.3(4)
S(31)-Ag(21)-S(29)	95.5(4)	P(8)-Ag(45)-Ag(26)	143.3(4)
S(17)-Ag(21)-S(29)	108.7(4)	S(1)-Ag(45)-Ag(26)	85.5(3)
P(5)-Ag(22)-S(17)	122.1(5)	S(30)-Ag(45)-Ag(26)	66.3(3)
P(5)-Ag(22)-S(8)	105.1(4)	S(12)-Ag(45)-Ag(26)	48.4(3)
S(17)-Ag(22)-S(8)	116.7(4)	P(16)-Ag(46)-S(35)	111.2(6)
P(5)-Ag(22)-S(24)	115.6(4)	P(16)-Ag(46)-S(36)	121.5(5)
S(17)-Ag(22)-S(24)	91.5(4)	S(35)-Ag(46)-S(36)	105.7(5)
S(8)-Ag(22)-S(24)	104.4(4)	P(16)-Ag(46)-S(34)	116.7(6)
P(5)-Ag(22)-Ag(4)	144.0(4)	S(35)-Ag(46)-S(34)	106.9(5)
S(17)-Ag(22)-Ag(4)	68.3(3)	S(36)-Ag(46)-S(34)	92.6(4)
S(8)-Ag(22)-Ag(4)	49.3(3)		

Symmetry transformations used to generate equivalent atoms:

#1 x,-y+2,z+1/2 #2 x,-y+2,z-1/2

Supplementary References

1. APEX II software suite, Bruker-AXS (2006).

2. Sheldrick, G. M. A short history of SHELX. *Acta Crystallogr.*, *A* **64**, 112–122 (2008).
